# The Effect of Sintering on Zirconia Manufactured via Suspension-Enclosing Projection Stereolithography for Dental Applications: An In Vitro Study

**DOI:** 10.3390/ma17010014

**Published:** 2023-12-19

**Authors:** Amit Unnadkat, Levi Kirby, Senthilguru Kulanthaivel, Oscar Rysavy, Akimasa Tsujimoto, Xuan Song, Erica C. Teixeira

**Affiliations:** 1 Department of General Dentistry, College of Dentistry, University of Tennessee Health Science Center, Memphis, TN 38163, USA; aunnadka@uthsc.edu; 2 Department of Industrial and Systems Engineering, The University of Iowa College of Engineering, Iowa City, IA 52242, USA; 3 Department of Operative Dentistry, The University of Iowa College of Dentistry and Dental Clinics, Iowa City, IA 52242, USA; senthilguru-kulanthaivel@uiowa.edu (S.K.); akimasa-tsujimoto@uiowa.edu (A.T.); 4 Division of Biostatistics and Computational Biology, The University of Iowa College of Dentistry and Dental Clinics, Iowa City, IA 52242, USA; 5 Department of Biostatistics, The University of Iowa College of Public Health, Iowa City, IA 52242, USA; 6 Department of Operative Dentistry, Aichi Gakuin University School of Dentistry, Chikusa-ku, Nagoya 464-8651, Aichi, Japan; 7 Department of General Dentistry, Creighton University School of Dentistry, Omaha, NE 68102, USA

**Keywords:** zirconia, dental restorations, additive manufacturing, stereolithography, sintering, flexural strength, material density, phase composition

## Abstract

Background: Zirconia is a widely used material in the dental industry due to its excellent mechanical and aesthetic properties. Recently, a new 3D printing process called suspension-enclosing projection stereolithography (SEPS) was introduced to fabricate zirconia dental restorations. However, the effect of the sintering time and temperature on the properties of zirconia produced via SEPS has not been fully investigated. Methods: Zirconia slurries were prepared with varying percentages of zirconia powders and 3D printing resins, and 5Y-TZP (5 mol% yttria-stabilized zirconia) (*n* = 40) and 3Y-TZP (3 mol% yttria-stabilized zirconia) (*n* = 40) bar specimens were fabricated via SEPS manufacturing. The specimens were sintered at different temperatures and dwell times, and their flexural strength, density, and phase composition were measured. The viscosity of the slurries was also measured. Statistical analysis was performed using Welch’s ANOVA and Kruskal–Wallis tests to evaluate the impact of the sintering conditions. Results: Significant differences in flexural strength (*p* < 0.01) were observed between the 5Y-TZP samples, with those sintered at 1530 °C for 120 min showing an average strength of 268.34 ± 44.66 MPa, compared to 174.16 ± 42.29 MPa for those sintered at 1450 °C for 120 min. In terms of density, significant differences (*p* < 0.01) were noted for the 3Y-TZP specimens, with an average density of 6.66 ± 0.49 g/cm^3^ for samples sintered at 1530 °C for 120 min, versus 5.75 ± 0.55 g/cm^3^ for those sintered at 1530 °C for 10 min. X-ray diffraction confirmed the presence of a predominantly tetragonal phase in both materials. Conclusions: Zirconia printed via SEPS manufacturing can be sintered at a higher temperature with shorter dwell times, thereby producing high density samples. Different sintering conditions can be used to fully sinter 3D-printed zirconia for potential dental applications.

## 1. Introduction

Additive manufacturing (AM), or 3D printing, has emerged as a powerful tool in the field of dentistry, enabling the fabrication of customized dental prostheses. Stereolithography (SLA) is the preferred additive manufacturing technique for dental applications because it provides the highest accuracy and resolution [1]. SLA-manufactured restorations can be influenced by a multitude of preprinting, printing, and post-printing factors such as reinforcing agent additions, printing orientation, layer thickness, resin type, build platform position, and post-curing parameters, all of which collectively impact the flexural strength, material consumption, print efficiency, and overall quality of printed parts [2].

Stereolithography (SLA) can be effectively used for ceramic fabrication, known as ceramic stereolithography (CSL) or lithography-based ceramic manufacturing (LCM), enabling the creation of fully dense ceramics such as alumina [3], zirconia, tricalcium phosphate [4], and bioactive glass, with properties comparable to those produced through traditional manufacturing techniques. Zirconia is increasingly preferred for dental restorations, especially in high occlusal load areas, due to its superior mechanical properties, aesthetic properties, sufficient radiopacity, and resistance to microbial adhesion [5]. Digital workflows are used to fabricate these restorations and heavily rely on specialized software for precision and efficiency [6,7]. In addition to their application in natural teeth restoration, digital workflows, particularly those utilizing CAD/CAM technologies, have expanded to include the fabrication of dental implant abutments [8].

Ceramic additive manufacturing relies on the processes of debinding and sintering to achieve the desired final properties of the material. Debinding, which involves the removal of the polymeric binder, is influenced by parameters such as component geometry, solvent temperature, particle size, pore size, and total solid content, and can involve multiple steps or specific techniques such as vacuum debinding to avoid defects and improve efficiency [9,10,11,12,13,14]. Following debinding, the sintering process optimizes the material’s mechanical strength, density, and overall integrity.

Zirconia, typically sintered between 1350 °C and 1600 °C, undergoes yttrium incorporation at high temperatures [15]. Sintering beyond 1600 °C may result in unwanted outcomes like increased porosity and excessive grain growth [16,17]. On the contrary, zirconia sintered below 1400 °C tends to exhibit inadequate mechanical and optical properties. These properties, together with microstructure, grain size and resistance to aging, are significantly influenced by alterations to the duration and temperature of the sintering process and the yttria content [18]. Research on milled zirconia, however, has yielded mixed results on the effects of sintering on the material’s properties. Some studies have found no significant effect of the sintering temperature or holding time on the phase transformation or the flexural strength [19] of zirconia specimens [20,21]. Yet others report that alterations to these parameters can induce a phase transformation and affect the mechanical properties of the material [18]. The discrepancies in these findings may be attributed to variations in the structure of the material used, the sintering parameters, or the phase transformation of the material’s structure [17].

Recent technological advancements in induction furnaces have enabled high-speed sintering protocols for partially sintered zirconia, contrasting with conventional sintering procedures that last several hours [22]. Despite these advancements, the sintering parameters for additively manufactured zirconia have not been extensively evaluated. A handful of studies that have explored this area have reported encouraging results, with flexural strength values ranging from approximately 200 to 1500 MPa for SLA- and DLP-manufactured zirconia [23,24,25,26,27]. However, this is generally lower than the flexural strength of milled zirconia, which typically falls within the range of 900 to 1200 MPa. The recent development of a support-free printing system known as suspension-enclosing projection stereolithography (SEPS) has further expanded the scope of 3D printing and allows the use of ceramic materials [28,29]. However, there is a need to investigate the suitability of SEPS-manufactured zirconia for dental prostheses and understand the effect of sintering parameters on the material’s mechanical properties [30].

The aim of this study was to evaluate the effect of the sintering time and temperature on the flexural strength and density of zirconia manufactured via SEPS. Hypothesizing that the sintering time and temperature significantly influence the flexural strength and density of zirconia, the study anticipated that variations in sintering parameters would lead to measurable differences in these properties. Conversely, the null hypothesis posits that these variations in sintering conditions will not have a significant impact on the flexural strength and density of the zirconia. In this context, slurries of powders with different zirconia yttria contents were prepared using SEPS and subjected to different sintering conditions. The significance of this study lies in the potential to manufacture fully sintered zirconia dental prostheses using the SEPS process. This would provide a cost-effective and efficient alternative to the traditional milling process, while also allowing customization and high precision in fabrication.

## 2. Materials and Methods

### 2.1. Materials

Two different zirconia powders, 3Y (Zpex, Tosoh, Tokyo, Japan) and 5Y (Zpex Smile, Tosoh, Tokyo, Japan), were used to prepare slurries with concentrations of 60 and 70 wt%, respectively. The photopolymer resins used included 20 wt% clear resin (FLGPCL04, Formlabs, Somerville, MA, USA) and 20 wt% basic white resin (Anycubic, Shenzhen, Guandong, China) for the 3Y suspension and 15 wt% of each for the 5Y suspension. After the initial mix, Solsperse 20000 (oxyalkylated amine, Lubrizol, Wickliffe, OH, USA) at 1.5 wt% was added to aid in the particle dispersion and suspension stability. Additionally, <0.5 g of 99% isopropyl alcohol was included as a diluent to achieve the required consistency. Mixing was performed manually until a uniform slurry was obtained. Post-mixing, the slurries were further processed in a ball mill. Each slurry was placed into a zirconia jar containing zirconia ball bearings and milled for 2 h at 300 RPM. Following milling, the slurries underwent a degassing process under a vacuum at −1.5 bar for three 15 min cycles, where a cessation of bubbling was noted.

### 2.2. Instruments

A SEPS printer [28,29] was used, together with a ball mill machine set at 300 RPM for 2 h, a rheometer (MCR-72, Anton Paar, GmbH, Graz, Austria) with shear rates from 0.01 to 1000 s^−1^, a UV curing chamber (LED UV λ 375–405 nm, XYZPrinting, Lake Forest, CA, USA) set for 10 min, an argon-filled tube furnace for debinding at 600 °C with a dwell time of 175 min, and a sintering furnace (Ceramill Therm 3, Amann Girrbach AG, Koblach, Austria) with a constant heating rate of 8 °C/min. Testing equipment included a universal testing machine (Proline Z005, Zwick Roell, Ulm, Germany) with a crosshead speed of 1 mm/min, an electronic balance with an accuracy of 0.001g, a Rigaku SmartLab diffractometer (Rigaku, Tokyo, Japan) with voltage and current set to 40 kV and 40 mA, and a Hitachi S-4800 scanning electron microscope (Hitachi High-Technologies, Tokyo, Japan).

### 2.3. Specimen Preparation Protocol

**Figure 1:**
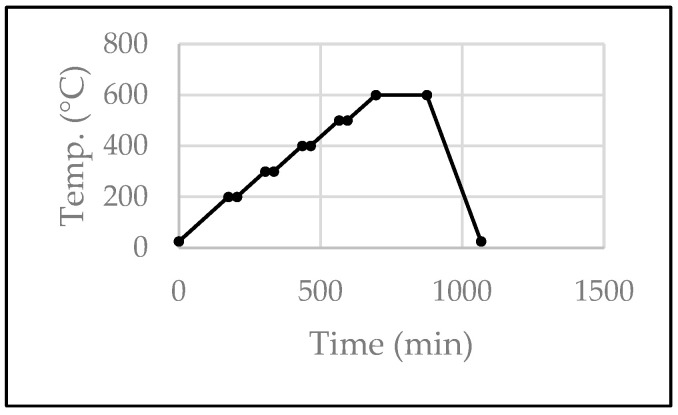
Debinding parameters.

**Figure 2:**
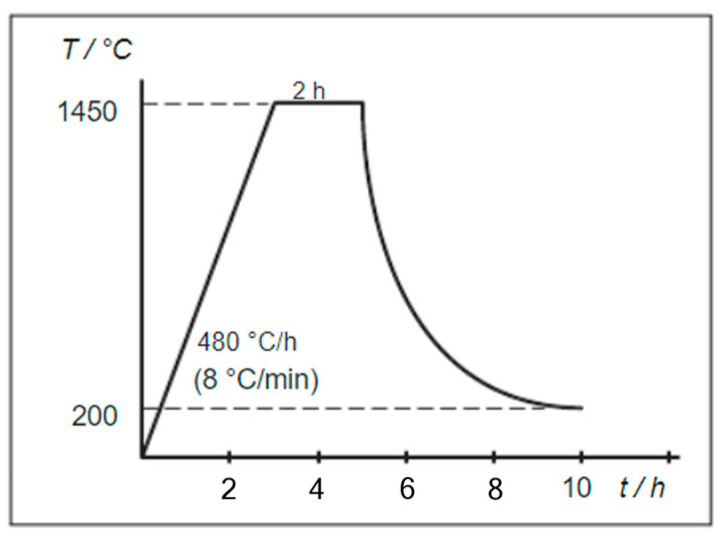
Sintering parameters (1450 °C, 2 h)—Amann Girrbach THERM 3 User Manual.

The pre-sintered dimensions of the bar (36.2 × 4.9 × 1.5 mm) were used to design an STL file using a Meshmixer (v3.5.474, Autodesk, San Rafael, CA, USA) with expected volumetric shrinkage post-sintering. Specimens were fabricated in groups varying in yttria content (3Y and 5Y), sintering time (10, 30, and 120 min), and sintering temperature (1450 °C and 1530 °C). Following the creation of ceramic suspensions and their ball milling for 2 h, the suspensions were used to manufacture bars via the SEPS process, which involved 28 layers at a 45 s exposure to achieve a curing depth of 62 μm and a final height of 1.5 mm. After UV curing, the specimens underwent debinding in a tube furnace at 600 °C with dwell times of 175 min (Figure 1), followed by sintering at the designated temperatures and for the holding times shown in Table 1 and Figure 2.

### 2.4. Testing

#### 2.4.1. Dimensional Accuracy and Flexural Strength Testing

Digital micrometry was employed to measure the dimensions of all of the sintered specimens with an accuracy of 0.01 mm. The flexural strength was determined at room temperature via three-point bending tests using a universal testing machine (Proline, Z005, Zwick Roell, Ulm, Germany). The specimens were subjected to a load cell capable of exerting a force up to 500 N, with a crosshead speed set at 1 mm/min. The flexural strength, denoted as σ, was calculated via the formula σ = 3FL/(2bd^2^), where F is the fracture load (N), L is the span between supports (mm), b denotes the width (mm), and d represents the thickness of the specimens (mm). All of the specimens were inspected after testing and two specimens were excluded due to visible printing defects.

#### 2.4.2. Density Testing

Archimedes’ principle was applied for the determination of zirconia sample densities. The dry sample mass (M) and the volume of water displaced (V) were quantified using an electronic balance with an accuracy of 0.001 g. Density values were calculated based on the ratio d = M/V.

#### 2.4.3. Crystal Structure Analysis

For the analysis of crystallographic phases, one specimen from each test group was selected for X-ray diffraction (XRD) examination. The samples were analyzed using a Rigaku SmartLab diffractometer, subjected to Cu Ka radiation, and the diffraction patterns were recorded within a 2θ range of 5 to 80° range at a scan rate of 5°/minute with an incremental step size of 0.01. The equipment was operative at a voltage of 40 kV and a current of 40 mA. Quantification of the monoclinic (m), tetragonal (t), and cubic (c) phases was performed using Rietveld analysis with TOPAS v4.0 software.

#### 2.4.4. Scanning Electron Microscopy (SEM) Analysis

One sample was randomly selected from each group for SEM analysis. The samples were sputter-coated with iridium prior to analysis. The SEM was performed using a Hitachi S-4800 scanning electron microscope with a 3 kV acceleration voltage applied to both the upper and lower detectors. The analysis was conducted in secondary electron mode under high vacuum conditions, allowing for detailed surface characterization. Magnifications of 400×, 2000×, and 4000× were used, and no backscattered or electron mapping was performed.

### 2.5. Statistical Analysis

The statistical analysis in this study was conducted using R, as cited by R Core Team (2022) [31]. Descriptive statistics were used to summarize the data. The Shapiro–Wilk test was used to assess the normality of the ANOVA residuals, a key assumption for ANOVA’s validity. Three of the ANOVA models used for the dimensional analysis failed this normality test. Consequently, these non-significant Welch’s ANOVA results were corroborated using the non-parametric Kruskal–Wallis test. Welch’s ANOVA was applied to discern differences in mean flexural strength and density between various sintering conditions for both the 3Y and 5Y materials. Pairwise Welch’s *t*-tests with the Holm adjustment identified groups with differing mean values in flexural strength and density. The study also explored the correlation between flexural strength and density.

## 3. Results

### 3.1. Rheological Properties—Ceramic Suspension

**Figure 3:**
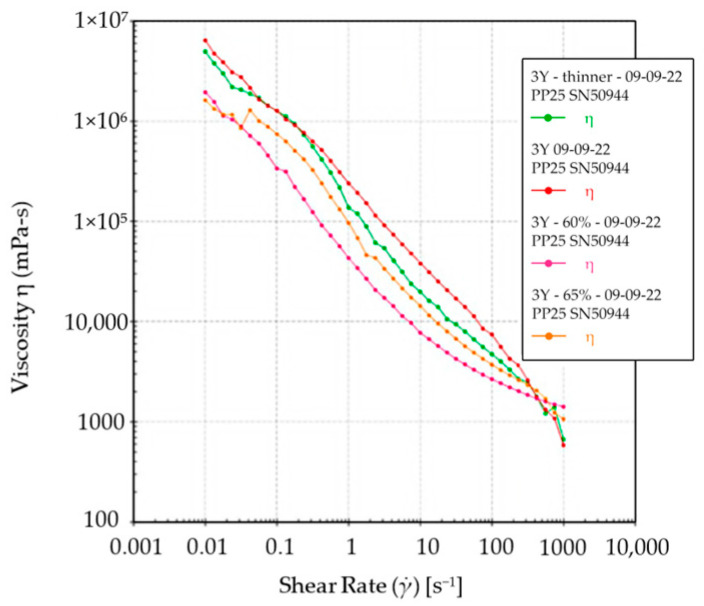
3Y Slurry thinning.

**Figure 4:**
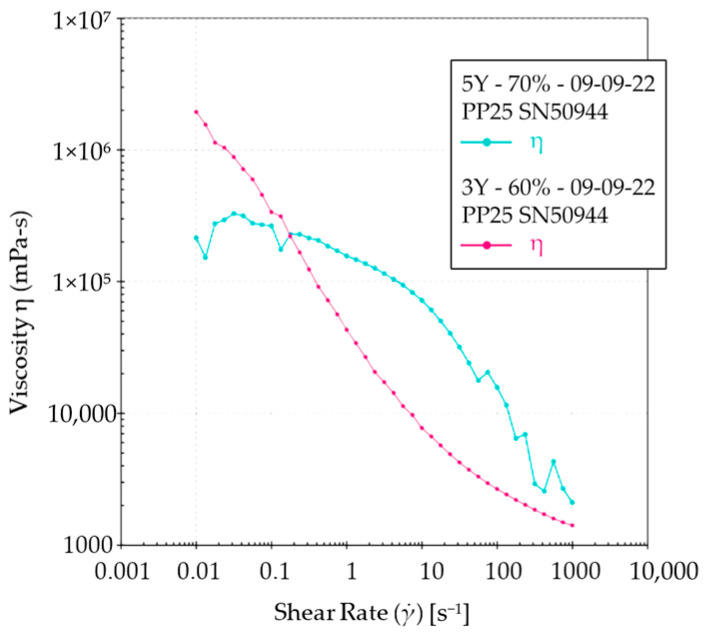
Comparison of 3Y and 5Y slurries.

The rheological properties of the ceramic suspension were analyzed to understand its flow behavior during SEPS printing. Both the 3Y and 5Y slurries (Figure 3 and Figure 4) exhibited shear-thinning behavior, where viscosity decreased as the shear rate increased. The 3Y slurries showed a more rapid decrease in viscosity compared to the 5Y slurry. Additionally, the flow curves indicated a high yield stress, which signifies the stress required to initiate the flow. These rheological properties make the slurries suitable for SEPS printing, ensuring easy flow and shape retention.

### 3.2. Physical Properties

#### 3.2.1. Dimensional Accuracy

The dimensions of the sintered samples were measured and compared between different sintering conditions. For both the 3Y and 5Y samples, minor variations in the mean values of length and width were observed across the different sintering conditions (Table 2 and Table 3). However, for the 3Y samples, the 1450 °C and 120 min group exhibited higher mean shrinkage in height compared to the 1530 °C and 120 min group. For the 5Y samples, no significant differences in dimensions were found, regardless of the temperature or time evaluated.

#### 3.2.2. Flexural Strength and Density

**Figure 5:**
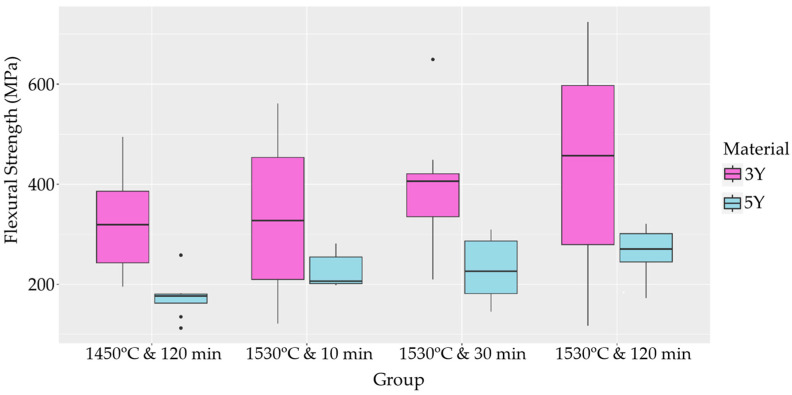
Boxplot analysis of sintered flexural strength.

**Figure 6:**
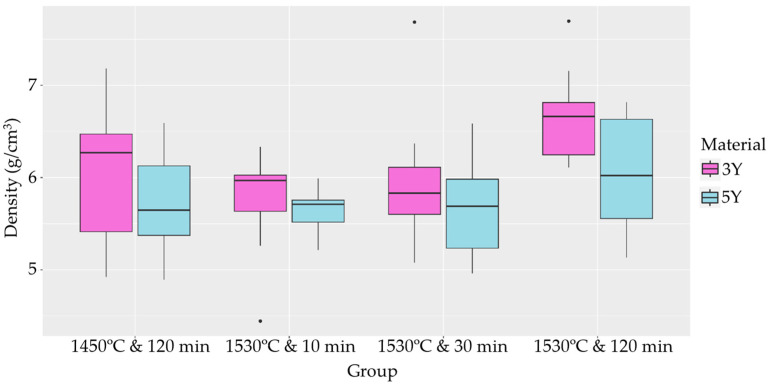
Boxplot analysis of sintered densities.

The flexural strength and density of the sintered samples were determined. Welch’s ANOVA revealed no significant differences in mean flexural strength between the different sintering conditions for the 3Y samples (Table 4). However, for the 5Y samples, significant differences in mean flexural strength were observed between the different sintering conditions (Table 5). Pairwise Welch’s *t*-tests with Holm adjustment identified specific groups with significantly different mean flexural strength values, as depicted in a boxplot (Figure 5). In terms of density, significant differences in mean density were observed between sintering conditions for the 3Y samples, while no significant differences were found for the 5Y samples, which is visually summarized in a density boxplot (Figure 6). Pearson’s product–moment correlation revealed no association between flexural strength and density.

#### 3.2.3. Phase Composition and Microstructure

**Figure 7:**
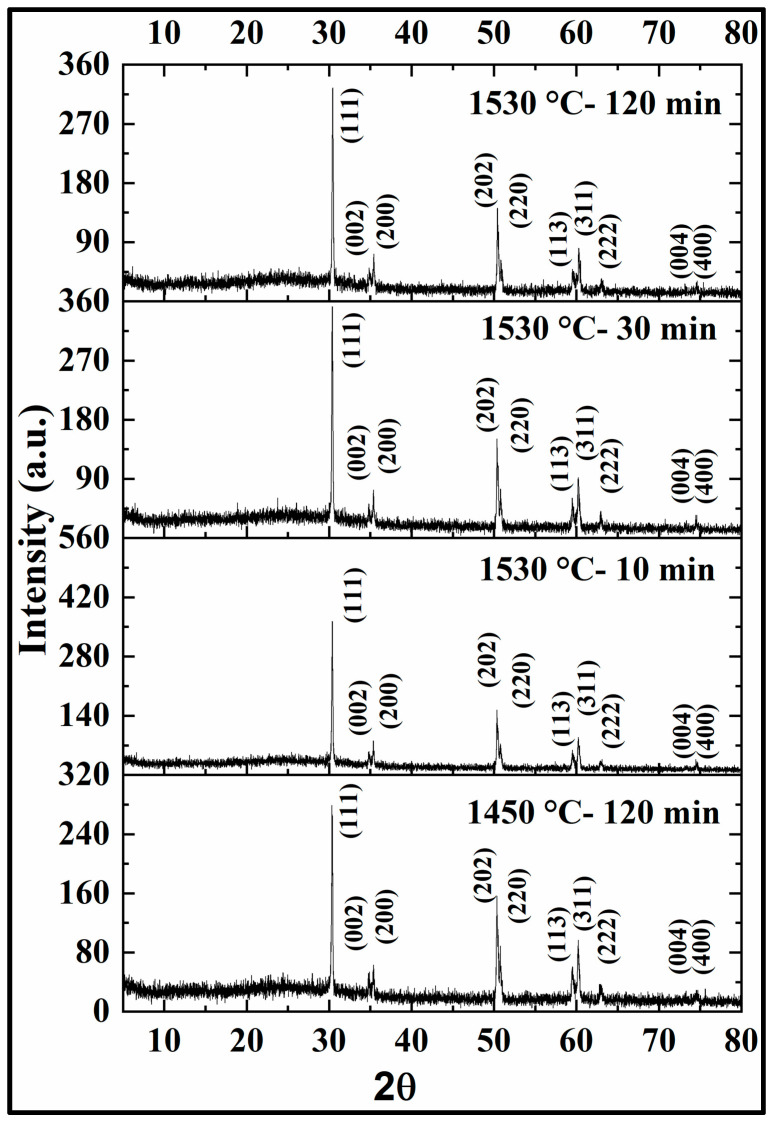
3Y-TZP XRD.

**Figure 8:**
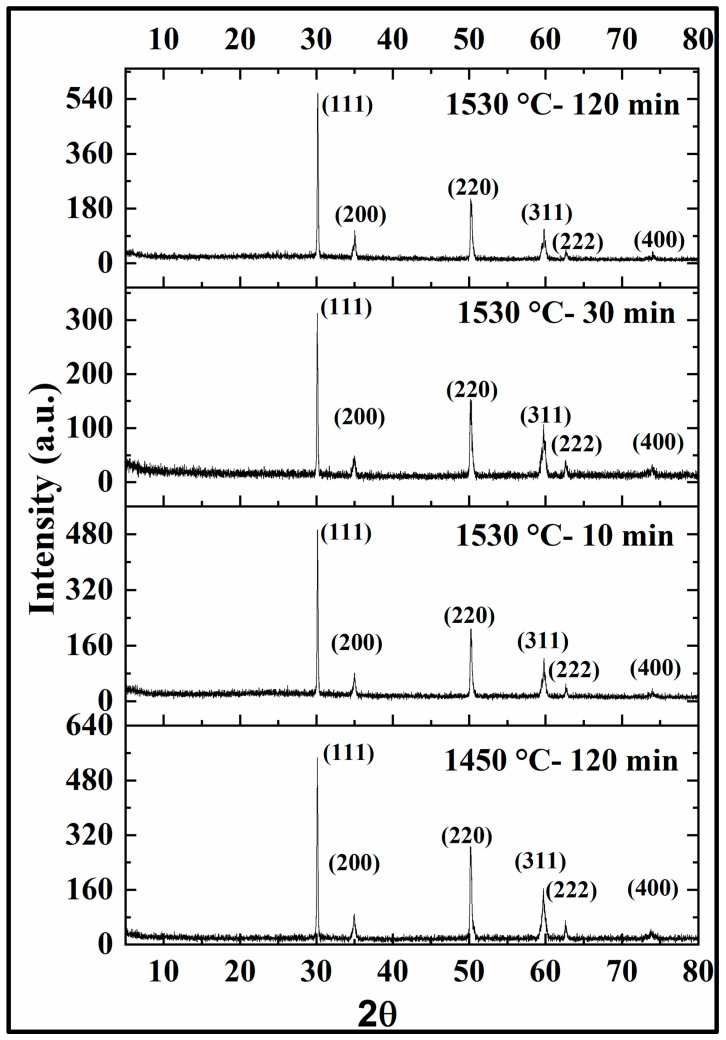
5Y-TZP XRD.

XRD is often employed to confirm the phase content of samples. In this study, XRD was performed to analyze the change in phase content of the zirconia samples with respect to the different sintering conditions. The XRD patterns of 3Y zirconia and 5Y zirconia under each sintering condition are presented in Figure 7 and Figure 8, respectively. The XRD patterns showed signature peaks of zirconia at the corresponding 2θ values, with the intensity of each peak measured in arbitrary units (a.u.). For 3Y (3Y-TZP partially stabilized zirconia), all four test groups showed similar patterns, with peaks at 30.5, 35, 35.5, 50.3, 51, 59.5, 60.5, 63.3, 73.3, and 74.5 2θ corresponding to the crystal planes 111, 002, 200, 202, 220, 113, 311, 222, 004, and 400, respectively. The intensity of these peaks varied slightly between the different test groups. For 5Y (5Y-TZP partially stabilized zirconia), peaks were also observed at 30.3, 35, 50.4, 60, and 62.5 2θ, corresponding to the crystal planes 200, 220, 311, 222, and 400, respectively.

Scanning electron microscopy (SEM) was used to examine the microstructure of 3Y and 5Y zirconia sintered at different temperatures and under different conditions. Images were obtained at magnifications of 400×, 2000×, and 4000×.

The SEM micrographs at a lower magnification showed a limited number of voids, with an overall dense structure for both the 3Y and 5Y samples.

**Figure 9:**
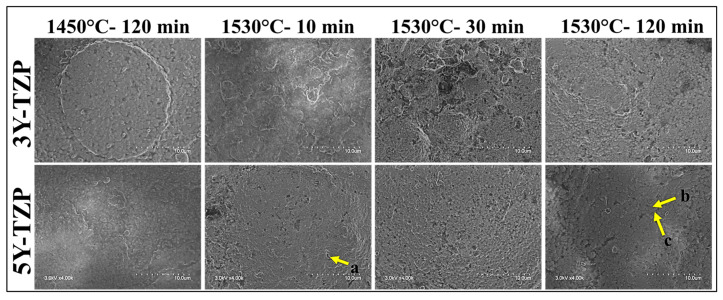
SEM micrographs (4000×) of the zirconia samples under different sintering conditions. The yellow arrows indicate: (**a**) void, (**b**) grain, and (**c**) grain boundary.

Upon closer inspection of the 5Y samples in the SEM (Figure 9), we detected an increase in the grain size with an increase in the sintering temperature and the holding time. As expected, the grain boundaries were also more evident with an increase in temperature and isothermal holding time. In the case of the 3Y samples, with an increase in the holding time from 10 min to 120 min, the grain size increased with prominent grain boundaries. Here, the lower sintering temperature of 1450 °C with a longer holding time of 120 min resulted in a larger grain size in comparison to the higher sintering temperature (1530 °C) with a shorter holding time.

## 4. Discussion

Currently, additively manufactured ceramics face shortcomings related to dimensional accuracy, mechanical properties, and aesthetics [30], which have limited their widespread adoption compared to subtractive methods. These limitations may be overcome by optimizing the factors affecting the material properties. These factors include raw material selection, printing parameters, and post-processing treatments [32,33]. To address these issues, it has been suggested that an optimized sintering protocol may improve both the dimensional accuracy and mechanical properties [30]. This study focused on examining the role of heat treatment as a key factor in addressing these challenges.

In this study, we assessed and compared four different sintering conditions for manufacturing zirconia specimens via suspension-enclosing projection stereolithography (SEPS). One of the most important factors in 3D printing is the rheological properties of the slurry. The rheological properties of the ceramic suspensions used for SEPS printing showed shear-thinning behavior, high viscosity at low shear rates, and high yield stress, which are desirable characteristics for the printing process [29]. The suspensions exhibited a decrease in viscosity as the shear rate increased, allowing them to flow easily and fill the desired shape. The high yield stress ensured proper flow initiation during printing.

X-ray diffraction (XRD) analysis of the sintered zirconia samples revealed that the sintering conditions did not significantly affect the crystalline phase composition, with all groups predominantly exhibiting a tetragonal phase. The peaks observed in the XRD patterns for 3Y-TZP and 5Y-TZP were consistent with those reported in previous studies [34,35]. Scanning electron microscopy (SEM) images showed relatively uniform and well-connected grains with some small pores and voids in the sintered 3Y samples. In contrast, the sintered 5Y samples displayed a more heterogeneous microstructure with a mixture of large and small grains, as well as areas of porosity. Several studies have reported an increase in grain size with an increase in the sintering temperature [36,37,38,39]. Moreover, Avles et al. (2022) observed a considerable grain growth with an increased holding time in the case of 3Y-TZP [40].

The debinding and sintering conditions have a significant impact on the properties of additively manufactured zirconia. This is due to the high polymer content in 3D-printed ZrO2 samples, which may lead to weaker binding forces between layers, consequently affecting the material’s flexural strength and overall integrity. In contrast to our one-step debinding process, Hyun Ji et al. (2021) demonstrated enhanced flexural strength with a multi-stage debinding, suggesting its potential benefits [41]. They employed a three-step debinding procedure based on insights into the role of internal stresses that emerge during the drying and debinding of 3D-printed ceramics. Such internal stresses have been attributed to layer separation and cracking, predominantly caused when solvents, residues, and polymers escape from the objects during these stages [41].

To counteract these challenges, Hyun Ji et al. (2021) examined different drying conditions for the post-cured green body and revealed significant differences in weight loss between drying methods. Specifically, while oven drying at 80 °C induced heightened internal stresses resulting in more defects, vacuum drying at 25 °C for 1 h was markedly effective in preventing such defect formation. This was primarily attributed to the slower and more controlled removal of the material under vacuum conditions, which ultimately minimized internal stress. [41] In our study, the specimens were also placed in a vacuum chamber at 20 °C, extending up to 24 h before post-processing in a controlled process. With an extended duration in the vacuum, our goal was to achieve consistent and gradual evaporation of residual solvents, thereby reducing potential defects.

Furthermore, based on TG-DTA analysis, Hyun Ji et al. identified a distinct weight loss point at 380 °C, leading to an optimized three-step debinding process for 3YSZ 3D-printed objects with holding times at 300 °C, 380 °C, and 700 °C. This approach allowed a more gradual and controlled removal of polymers, resulting in minimal defects and a high-density sintered body with a notable flexural strength of 1002.5 MPa when sintered at 1450 °C. In our study, high-density specimens were consistently achieved, as demonstrated by both SEM evaluations and density measurements derived through Archimedes’ method. These densities closely align with the manufacturer’s specified density for TOSOH Zpex^®^ and Zpex Smile^®^ zirconia powders, which is 6.08 g/cm^3^ for the sintered body [42]. Additionally, our findings bear a close resemblance to the densities observed by Hyun Ji et al., albeit achieved under different procedural conditions.

The sintering protocol can also influence the formation of grains and grain boundaries. Increasing the sintering temperature and holding time can lead to enhanced grain growth, reduced porosity, and better crystalline phase stability, resulting in improved mechanical properties. At higher sintering temperatures, the mobility of atoms is increased, facilitating the coalescence of small grains into larger ones [17]. Additionally, higher temperatures accelerate the sintering rate, leading to denser microstructures and fewer defects. In our study, a higher sintering temperature showed a significant difference in mean flexural strength between the sintering conditions for the 5Y material. The group sintered at 1530 °C for 120 min exhibited higher flexural strength compared to the group sintered at 1450 °C for 120 min. However, no significant difference was observed in mean flexural strength between the 3Y groups.

The significant difference in mean flexural strength observed between the sintering conditions for the 5Y material can be attributed to the specific sintering parameters used. The longer sintering time of 120 min and the higher sintering temperature of 1530 °C resulted in a denser microstructure and significantly higher flexural strength. However, the lack of significant differences in mean flexural strength between the 3Y groups may be due to the inherent differences in the zirconia composition and the interplay between sintering parameters and material properties.

Regarding density, a significant difference was observed between the sintering conditions for the 3Y material, with the group sintered at 1530 °C for 120 min displaying a higher mean density compared to the group sintered at 1530 °C for 10 min.

The effect of sintering on the density was significant for the 3Y material at 1530 °C for 10 min compared to 120 min, with longer sintering times leading to a higher mean density. The optimal sintering conditions may vary depending on the yttria content and other material properties [43]. The 5Y material groups did not show significant differences in mean density.

The dimensional accuracy of additively manufactured dental ceramics has been investigated by several authors. Zirconia has the potential to achieve superior dimensional accuracy, as shown by Ferrini et al. [44], who documented milled zirconia’s notable marginal fit compared to lithium disilicate and composite crowns. Within the range investigated, our observations revealed that the sintering time and temperature are not significant determinants of material shrinkage for printing via SEPS. Furthermore, existing studies indicate that numerous factors, including the build angle, laminating direction, and support configuration, significantly affect the dimensional accuracy [45,46,47] together with light scattering and a reduced depth of cure [48]. Particularly, material properties can significantly impact the printing accuracy. Jang et al. examined the effects of zirconia’s volume fraction (Vv) on the quality of 3D-printed constructs [49]. Their research showed that as zirconia’s Vv increases, the Zr-based suspension becomes less flowable. The optimal threshold was identified at a Vv of 58 vol% (or 89 wt%), beyond which reliable printing becomes challenging. Additionally, a rise in Vv resulted in a reduced cure depth for the suspensions, which could introduce inaccuracies.

The distinct optical properties of zirconia further complicate this dynamic, as they cause a pronounced light-scattering effect and a refractive index that is 20–27% higher than other ceramics, like silica and alumina [50]. While these properties are inherent to zirconia, they can interfere with the polymerization process as light scattering limits the depth of cure [51].

The higher mean shrinkage in height for the 3Y samples sintered at 1450 °C for 120 min, compared to those at 1530 °C, aligns with the findings of Unikovsky et al. [47]. Their study emphasized that a 45° angulation with support structures could enhance dimensional accuracy. Notably, specimens from the same study angled at 45° or parallel to the load direction demonstrated superior axial load resistance, whereas the 90° specimens exhibited the highest mean flexural strength. Object placement on the build platform has also been shown to affect accuracy, as centrally located objects tend to be less susceptible to inaccuracies compared to peripheral placement [47]. Additionally, post-curing is believed to eliminate anisotropy in resins with pigments that permit UV light penetration [48].

While our research has provided valuable insights into sintering parameters, key limitations include the limited exploration of ceramic suspension properties and the constrained scope of the study to specific sintering conditions, which may not encompass the full spectrum of potential parameters. Additionally, the research mainly addressed material properties, and further investigations are needed to evaluate clinical applicability.

## 5. Conclusions

Our findings suggest that the ceramic suspensions used in this study show promise for SEPS printing as indicated by their shear-thinning behavior and high yield stress. However, we note that these results are preliminary and may be specific to the conditions of the given design. While variations in dimensions between the different sintering conditions were minimal, we observed statistically significant height shrinkage differences in the 3Y material. Within the scope of the tested parameters, our data indicate that the sintering time and temperature have a more pronounced impact on the flexural strength of 5Y zirconia compared to 3Y zirconia. However, further research is needed to fully understand these differences. The variations in density suggest the sensitivity of the 3Y zirconia to sintering parameters. This underscores the potential importance of optimizing the sintering process in SEPS in achieving the desired material properties.

While this study focused on the rheological properties, dimensions, flexural strength, and density, it is important to note that other mechanical properties, such as hardness and fracture toughness, warrant further investigation. Furthermore, the formulation of suspensions capable of consistent performance under varying processing conditions may better align the slurry with the printing process to improve mechanical properties and allow uniform shrinkage. Future studies will consider the range of factors affecting printed restorations, including cell compatibility and stability under aging conditions. Understanding the comprehensive mechanical behavior of additively manufactured zirconia will contribute to its successful implementation in clinical settings.

## Figures and Tables

**Table 1:** Debinding and Sintering Paremeters—Experimental groups.

Group *n* = 10	Debinding Temperature	Debinding Dwell Time	Sintering Temperature	Sintering Dwell Time	Total Time
1	600 °C	175 min	1450 °C	120 min	24.03 h
2	600 °C	175 min	1530 °C	10 min	22.56 h
3	600 °C	175 min	1530 °C	30 min	22.81 h
4	600 °C	175 min	1530 °C	120 min	24.13 h

**Table 2:** Sample dimensions for the 3Y group after heat treatment (debinding and sintering).

Treatment (*n* = 10)	L (mm) ^1^	W (mm) ^1^	H (mm) ^1^
1450 °C & 120 min	21.18 ± 0.18	3.15 ± 0.16	0.78 ± 0.04
1530 °C & 10 min	21.22 ± 0.20	3.13 ± 0.13	0.84 ± 0.08
1530 °C & 30 min	21.29 ± 0.26	3.21 ± 0.16	0.86 ± 0.08
1530 °C & 120 min	21.23 ± 0.14	3.18 ± 0.11	0.89 ± 0.05

^1^ Mean ± SD.

**Table 3:** Sample dimensions for the 5Y group after heat treatment (debinding and sintering).

Treatment (*n* = 10)	L (mm) ^1^	W (mm) ^1^	H (mm) ^1^
1450 °C & 120 min	23.55 ± 0.07	3.39 ± 0.12	0.91 ± 0.07
1530 °C & 10 min	23.77 ± 0.28	3.49 ± 0.12	0.98 ± 0.09
1530 °C & 30 min	23.56 ± 0.19	3.46 ± 0.19	0.93 ± 0.06
1530 °C & 120 min	23.59 ± 0.21	3.44 ± 0.05	0.96 ± 0.09

^1^ Mean ± SD.

**Table 4:** 3Y flexural strength and density.

Treatment	Flexural Strength (MPa, *n* = 10) ^1^	Density (g/cm^3^, *n* = 10) ^1^
1450 °C & 120 min	324.87 ± 101.52	6.02 ± 0.73
1530 °C & 10 min	334.91 ± 160.14	5.75 ± 0.55
1530 °C & 30 min	392.90 ± 116.83	5.98 ± 0.70
1530 °C & 120 min	435.89 ± 204.10	6.66 ± 0.49

^1^ Mean ± SD.

**Table 5:** 5Y flexural strength and density.

Treatment	Flexural Strength (MPa, *n* = 10) ^1^	Density (g/cm^3^, *n* = 10) ^1^
1450 °C & 120 min ^2^	174.16 ± 42.29	5.71 ± 0.56
1530 °C & 10 min	224.78 ± 34.21	5.66 ± 0.24
1530 °C & 30 min	228.47 ± 62.42	5.71 ± 0.57
1530 °C & 120 min	268.34 ± 44.66	6.02 ± 0.65

^1^ Mean ± SD; ^2^ missing 2 flexural strength measurements.

## Data Availability

New data were created and analyzed during this study. These datasets are not publicly available at this time, but can be obtained from the corresponding author, upon reasonable request.

## References

[1] Khanlar L.N., Salazar Rios A., Tahmaseb A., Zandinejad A. (2021). Additive Manufacturing of Zirconia Ceramic and Its Application in Clinical Dentistry: A Review. Dent. J..

[2] Song X., Chen Y., Lee T.W., Wu S., Cheng L. (2015). Ceramic fabrication using Mask-Image-Projection-based Stereolithography integrated with tape-casting. J. Manuf. Process..

[3] Gruber S. (2013). Lithography-Based Additive Manufacturing of Alumina Parts. Ph.D. Thesis.

[4] Mitteramskogler G. (2011). Generative Fertigung von Bauteilen aus TCP. Master’s Thesis.

[5] Baysal N., Tuğba Kalyoncuoğlu Ü., Ayyıldız S. (2022). Mechanical Properties and Bond Strength of Additively Manufactured and Milled Dental Zirconia: A Pilot Study. J. Prosthodont..

[6] Cabanes-Gumbau G., Soto-Peñaloza D., Peñarrocha-Diago M., Peñarrocha-Diago M. (2019). Analogical and digital workflow in the design and preparation of the emergence profile of biologically oriented preparation technique (BOPT) crowns over implants in the working model. J. Clin. Med..

[7] Adobes Martin M., Lipani E., Bernes Martinez L., Alvarado Lorenzo A., Aiuto R., Garcovich D. (2022). Reliability of Tooth Width Measurements Delivered by the Clin-Check Pro 6.0 Software on Digital Casts: A Cross-Sectional Study. Int. J. Environ. Res. Public Health.

[8] Menchini-Fabris G.B., Crespi R., Toti P., Crespi G., Rubino L., Covani U. (2020). A 3-year retrospective study of fresh socket implants: CAD/CAM customized healing abutment vs. cover screws. Int. J. Comput. Dent..

[9] Hwang K., Hsieh Y. (1996). Comparative study of pore structure evolution during solvent and thermal debinding of powder injection molded parts. Metall. Mater. Trans. A.

[10] Shivashankar T.S., German R.M. (1999). Effective length scale for predicting solvent-debinding times of components produced by powder injection molding. J. Am. Ceram. Soc..

[11] Omar M., Ibrahim R., Sidik M., Mustapha M., Mohamad M. (2003). Rapid debinding of 316L stainless steel injection moulded component. J. Mater. Process. Technol..

[12] Yang W.-W., Yang K.-Y., Wang M.-C., Hon M.-H. (2003). Solvent debinding mechanism for alumina injection molded compacts with water-soluble binders. Ceram. Int..

[13] Oliveira R.V., Soldi V., Fredel M.C., Pires A.T. (2005). Ceramic injection moulding: Influence of specimen dimensions and temperature on solvent debinding kinetics. J. Mater. Process. Technol..

[14] Krauss V.A., Oliveira A., Klein A., Al-Qureshi H., Fredel M. (2007). A model for PEG removal from alumina injection moulded parts by solvent debinding. J. Mater. Process. Technol..

[15] Luthardt R.G., Holzhüter M., Sandkuhl O., Herold V., Schnapp J.D., Kuhlisch E., Walter M. (2002). Reliability and Properties of Ground Y-TZP-Zirconia Ceramics. J. Dent. Res..

[16] Jang G., Kim H., Choe H., Son M. (2011). Fracture strength and mechanism of dental ceramic crown with zirconia thickness. Procedia Eng..

[17] Stawarczyk B., Özcan M., Hallmann L., Ender A., Mehl A., Hämmerlet C.H. (2013). The effect of zirconia sintering temperature on flexural strength, grain size, and contrast ratio. Clin. Oral Investig..

[18] Inokoshi M., Zhang F., De Munck J., Minakuchi S., Naert I., Vleugels J., Van Meerbeek B., Vanmeensel K. (2014). Influence of sintering conditions on low-temperature degradation of dental zirconia. Dent. Mater..

[19] Ebeid K., Wille S., Hamdy A., Salah T., El-Etreby A., Kern M. (2014). Effect of changes in sintering parameters on monolithic translucent zirconia. Dent. Mater..

[20] Öztürk C., Can G. (2019). Effect of Sintering Parameters on the Mechanical Properties of Monolithic Zirconia. J. Dent. Res. Dent. Clin. Dent. Prospect..

[21] Hjerppe J., Vallittu P.K., Fröberg K., Lassila L.V. (2009). Effect of sintering time on biaxial strength of zirconium dioxide. Dent. Mater..

[22] Uçar Y., Aysan Meriç İ., Ekren O. (2019). Layered manufacturing of dental ceramics: Fracture mechanics, microstructure, and elemental composition of lithography-sintered ceramic. J. Prosthodont..

[23] Lian Q., Sui W., Wu X., Yang F., Yang S. (2018). Additive manufacturing of ZrO ceramic dental bridges by stereolithography. Rapid Prototyp. J..

[24] Li R., Wang Y., Hu M., Wang Y., Xv Y., Liu Y., Sun Y. (2019). Strength and Adaptation of Stereolithography-Fabricated Zirconia Dental Crowns: An In Vitro Study. Int. J. Prosthodont..

[25] Revilla-León M., Al-Haj Husain N., Ceballos L., Özcan M. (2021). Flexural strength and Weibull characteristics of stereolithography additive manufactured versus milled zirconia. J. Prosthet. Dent..

[26] Revilla-León M., Al-Haj Husain N., Barmak A.B., Pérez-López J., Raigrodski A.J., Özcan M. (2022). Chemical Composition and Flexural Strength Discrepancies Between Milled and Lithography-Based Additively Manufactured Zirconia. J. Prosthodont..

[27] Zandinejad A., Das O., Barmak A.B., Kuttolamadom M., Revilla-León M. (2022). The Flexural Strength and Flexural Modulus of Stereolithography Additively Manufactured Zirconia with Different Porosities. J. Prosthodont..

[28] He L., Song X. (2018). Supportability of a High-Yield-Stress Slurry in a New Stereolithography-Based Ceramic Fabrication Process. JOM.

[29] He L., Fei F., Wang W., Song X. (2019). Support-Free Ceramic Stereolithography of Complex Overhanging Structures Based on an Elasto-viscoplastic Suspension Feedstock. ACS Appl. Mater. Interfaces.

[30] Li H., Song L., Sun J., Ma J., Shen Z. (2020). Stereolithography-fabricated zirconia dental prostheses: Concerns based on clinical requirements. Adv. Appl. Ceram..

[31] Team, R.C. (2022). R: A Language and Environment for Statistical Computing. https://www.R-project.org/.

[32] Chavez L.A., Ibave P., Wilburn B., Alexander D., Stewart C., Wicker R., Lin Y. (2020). The Influence of Printing Parameters, Post-Processing, and Testing Conditions on the Properties of Binder Jetting Additive Manufactured Functional Ceramics. Ceramics.

[33] Abualsaud R., Alalawi H. (2022). Fit, Precision, and Trueness of 3D-Printed Zirconia Crowns Compared to Milled Counterparts. Dent. J..

[34] Fonseca Y.R., Elias C.N., Monteiro S.N., Dos Santos H.E.S., Dos Santos C. (2019). Modeling of the Influence of Chemical Composition, Sintering Temperature, Density, and Thickness in the Light Transmittance of Four Zirconia Dental Prostheses. Materials.

[35] Zhang F., Reveron H., Spies B.C., Van Meerbeek B., Chevalier J. (2019). Trade-off between fracture resistance and translucency of zirconia and lithium-disilicate glass ceramics for monolithic restorations. Acta Biomater..

[36] Chen F., Wu J.-M., Wu H.-Q., Chen Y., Li C.-H., Shi Y.-S. (2018). Microstructure and mechanical properties of 3Y-TZP dental ceramics fabricated by selective laser sintering combined with cold isostatic pressing. Int. J. Lightweight Mater. Manuf..

[37] Denry I., Abdelaal M., Dawson D.V., Holloway J.A., Kelly J.R. (2021). Effect of crystalline phase assemblage on reliability of 3Y-TZP. J. Prosthet. Dent..

[38] Tong H., Tanaka C.B., Kaizer M.R., Zhang Y. (2016). Characterization of three commercial Y-TZP ceramics produced for their high-translucency, high-strength and high-surface area. Ceram. Int..

[39] Jansen J.U., Lümkemann N., Letz I., Pfefferle R., Sener B., Stawarczyk B. (2019). Impact of high-speed sintering on translucency, phase content, grain sizes, and flexural strength of 3Y-TZP and 4Y-TZP zirconia materials. J. Prosthet. Dent..

[40] Alves M.F.R.P., de Campos L.Q.B., Simba B.G., da Silva C.R.M., Strecker K., dos Santos C. (2022). Microstructural Characteristics of 3Y-TZP Ceramics and Their Effects on the Flexural Strength. Ceramics.

[41] Ji S.H., Kim D.S., Park M.S., Yun J.S. (2021). Sintering process optimization for 3YSZ ceramic 3D-printed objects manufactured by stereolithography. Nanomaterials.

[42] TOSOH (2023). Ceramic Materials—Zirconia. https://www.tosoheurope.com/our-products/ceramic-materials.

[43] Kulyk V., Duriagina Z., Kostryzhev A., Vasyliv B., Marenych O. (2022). Effects of Sintering Temperature and Yttria Content on Microstructure, Phase Balance, Fracture Surface Morphology, and Strength of Yttria-Stabilized Zirconia. Appl. Sci..

[44] Ferrini F., Paolone G., Di Domenico G.L., Pagani N., Gherlone E.F. (2023). SEM Evaluation of the Marginal Accuracy of Zirconia, Lithium Disilicate, and Composite Single Crowns Created by CAD/CAM Method: Comparative Analysis of Different Materials. Materials.

[45] Alharbi N., Osman R.B., Wismeijer D. (2016). Factors Influencing the Dimensional Accuracy of 3D-Printed Full-Coverage Dental Restorations Using Stereolithography Technology. Int. J. Prosthodont..

[46] Osman R.B., Alharbi N., Wismeijer D. (2017). Build Angle: Does It Influence the Accuracy of 3D-Printed Dental Restorations Using Digital Light-Processing Technology?. Int. J. Prosthodont..

[47] Unkovskiy A., Bui PH B., Schille C., Geis-Gerstorfer J., Huettig F., Spintzyk S. (2018). Objects build orientation, positioning, and curing influence dimensional accuracy and flexural properties of stereolithographically printed resin. Dent. Mater..

[48] Monzón M., Ortega Z., Hernández A., Paz R., Ortega F. (2017). Anisotropy of photopolymer parts made by digital light processing. Materials.

[49] Jang K.-J., Kang J.-H., Fisher J.G., Park S.-W. (2019). Effect of the volume fraction of zirconia suspensions on the microstructure and physical properties of products produced by additive manufacturing. Dent. Mater..

[50] Mitteramskogler G., Gmeiner R., Felzmann R., Gruber S., Hofstetter C., Stampfl J., Ebert J., Wachter W., Laubersheimer J. (2014). Light curing strategies for lithography-based additive manufacturing of customized ceramics. Addit. Manuf..

[51] Hinczewski C., Corbel S., Chartier T. (1998). Ceramic suspensions suitable for stereolithography. J. Eur. Ceram. Soc..

